# Wheat germ cell-free system-based production of hemagglutinin-neuraminidase glycoprotein of human parainfluenza virus type 3 for generation and characterization of monoclonal antibody

**DOI:** 10.3389/fmicb.2014.00208

**Published:** 2014-05-13

**Authors:** Satoko Matsunaga, Shiho Kawakami, Izumi Matsuo, Akiko Okayama, Hiroyuki Tsukagoshi, Ayumi Kudoh, Yuki Matsushima, Hideaki Shimizu, Nobuhiko Okabe, Hisashi Hirano, Naoki Yamamoto, Hirokazu Kimura, Akihide Ryo

**Affiliations:** ^1^Department of Microbiology, Yokohama City University School of MedicineKanagawa, Japan; ^2^Proteome Analysis Center, Yokohama City University School of MedicineKanagawa, Japan; ^3^Gunma Prefectural Institute of Public Health and Environmental SciencesGunma, Japan; ^4^Kawasaki City Health and Safety Research CenterKanagawa, Japan; ^5^Department of Microbiology, Yong Loo Lin School of Medicine, National University of SingaporeSingapore; ^6^Infectious Disease Surveillance Center, National Institute of Infectious DiseasesTokyo, Japan

**Keywords:** human parainfluenza virus 3, monoclonal antibody, cell-free protein synthesis, proteomics

## Abstract

Human parainfluenza virus 3 (HPIV3) commonly causes respiratory disorders in infants and young children. Monoclonal antibodies (MAbs) have been produced to several components of HPIV3 and commercially available. However, the utility of these antibodies for several immunological and proteomic assays for understanding the nature of HPIV3 infection remain to be characterized. Herein, we report the development and characterization of MAbs against hemagglutinin-neuraminidase (HN) of HPIV3. A recombinant full-length HPIV3-HN was successfully synthesized using the wheat-germ cell-free protein production system. After immunization and cell fusion, 36 mouse hybridomas producing MAbs to HPIV3-HN were established. The MAbs obtained were fully characterized using ELISA, immunoblotting, and immunofluorescent analyses. Of the MAbs tested, single clone was found to be applicable in both flow cytometry and immunoprecipitation procedures. By utilizing the antibody, we identified HPIV3-HN binding host proteins via immunoprecipitation-based mass spectrometry analysis. The newly-developed MAbs could thus be a valuable tool for the study of HPIV3 infection as well as the several diagnostic tests of this virus.

## INTRODUCTION

Human parainfluenza viruses (HPIVs) are major causes of lower respiratory infections in infants, young children, the immunocompromised, the chronically ill, and the elderly (Glezen et al., 1984; Counihan et al., 2001; Weinberg et al., 2009). HPIVs belong to the Paramyxoviridae family of medium-sized enveloped viruses and their genomes are organized on a single negative-sense strand of RNA. Of the four predominant serotypes of HPIV, the HPIV3 is the most frequently detected in viral infections in respiratory tracts. In fact, HPIV3 is second only to respiratory syncytial virus (RSV) as a cause of pneumonia and bronchiolitis in infants and young children. The HPIV3 genome contains approximately 15,000 nucleotides encoding at least six common structural proteins (3′-N-P-C-M-F-HN-L-5′; Storey et al., 1984; Tashiro and Homma, 1985; Wechsler et al., 1985). The two envelope glycoproteins, hemagglutinin-neuraminidase (HN) and fusion (F), are necessary for viral entry, cell-fusion and syncytium formation (Horvath et al., 1992; Hu et al., 1992; Takimoto et al., 2002; Porotto et al., 2012).

The HN protein is found on the lipid envelope of HPIVs, where it likely exists as a tetramer. HN is important for HPIV3 infection of host cells because it functions in virus-host cell attachment via sialic acid receptors and in virus release from cells with its neuraminidase activity (Huberman et al., 1995; Porotto et al., 2001; Chu et al., 2013). HN can be recognized by the host immune system and antibodies against epitopes within HN can neutralize its activity through the inhibition of the function and/or activity of either hemagglutinin or neuraminidase. Therefore, characterization of the protein structure and function of HN is of great importance for the understanding of HPIV3 infection and the host immunity against this virus.

Polyclonal antibodies and monoclonal antibodies (MAbs) against HPIV3 antigens have previously been generated, and animal antiserum to HPIV3 HN is also commercially available. However, the antibodies demonstrated cross-reactivity to other HPIV family viruses and exhibited relatively high non-specific background staining in immunoassays (Goswami and Russell, 1983; Waner et al., 1985). MAbs have been also produced to several components of HPIV3 (van Wyke Coelingh et al., 1985; Rydbeck et al., 1986). Although these commercially available MAbs have been shown to be specific, the utility of these antibodies for several immunological and/or proteomic analyses for understanding the nature of HPIV3 infection have not been fully delineated.

In our current study, we utilized the innovative wheat germ cell-free protein production system to generate the antigen protein. The main advantage of the cell-free protein system is the synthesis of proteins that are properly folded and that possess biological activity because the proteins are expressed in a eukaryotic cell system. Moreover, this system is capable of producing toxic proteins, such as viral antigens, that cause severe cytotoxicity or interference with host cellular physiology. By utilizing HPIV3-HN protein synthesized by the wheat cell-free system, we established multiple hybridoma clones producing MAbs that specifically targeted the viral antigen and were applicable for several immunoassays. Furthermore, we used the MAbs in proteomic analyses for identifying host proteins that potentially act as HN binding partners.

## MATERIALS AND METHODS

### CONSTRUCTION OF WHEAT GERM CELL-FREE EXPRESSION VECTOR

Amplification of HN fragment from HPIV3 (Strain C243) genome was performed with the following primers: forward (5′-AGGAGTAAAGTTACGCAATCCAA) and reverse (5′-ATATTTCCCTTTTGTCTATTGTCTG). For producing the expression vector of wheat germ cell-free system, The HN open-reading frame was amplified by PCR using the forward primer (5′-GAGAGGATCCCATGGAATACTGGAAGCAT) and reverse primer (5′-GAGAGCGGCCGCTTAACTGCAGCTTTTTGGA). The amplified fragment was subcloned using BamH I and Not I into pEU-His or pEU-bls-S1 (bls; biotin ligase site) vectors. Biotinylated HN mutants were generated using the PrimeSTAR Mutagenesis Basal kit (TakaraBio, Otsu, Japan) according to the manufacturer’s instructions.

HN protein fragments comprising CT, TM, and stalk regions were generated by the wheat cell-free system based on the template cDNA amplified from clinical isolates of HPIV1 (GeneBank No. JQ901977; Beck et al., 2012), HPIV2 (GeneBank No. AF533010; Skiadopoulos et al., 2003), and Mumps (GeneBank No. AB699704; Momoki, 2013) using following primer sets. HPIV1: (5′-ATGCTTATACTCTGGAGTCAAGA) and (5′-TCTAGCAAAACRTGAAGTTGAG); HPIV2: (5′-AAAAACCTA-AAATAAGCACGAA); and (5′-CCATTCTGGCCTATATYATAAT), Mumps: (5′-TTACTTATAAGACTGCGGTGC) and (5′-CTTGCA-ATGAGTTCTACTCTGA). Synthetic cDNA encoding the CT, TM and stalk regions of Sendai virus (UniProtKB entry no. P04853; Miura et al., 1985) was generated by Operon Biotechnologies (Huntsville, WI, USA). The amplified fragments were subcloned into pEU-bls-S1 vectors using In-fusion cloning system (TakaraBio) according to the manufacturer’s instructions.

### CELL-FREE PROTEIN SYNTHESIS AND PURIFICATION

*In vitro* transcription and cell-free protein synthesis were performed as described (Takai and Endo, 2010; Takai et al., 2010). For cell-free protein synthesis, the ENDEXT Wheat Germ Expression S Kit (CellFree Sciences, Yokohama, Japan) was used according to the manufacturer’s instructions for the bilayer translation method. GST fusion and Biotinylated proteins were produced as previously described (Sawasaki et al., 2008; Takahashi et al., 2012). His-HPIV3-HN protein, used for the generation of hybridoma, was synthesized using the robotic synthesizer (Protemist XE; CellFree Sciences) according to the manufacturer’s instructions. The cell-free translation reaction mixture (15 ml) was separated into soluble and insoluble fractions by centrifugation at 15,000 rpm for 15 min. The insoluble fraction was lysed by the addition of 8 M Urea at room temperature for 6 h, then mixed with Ni-sepharose High Performance beads (GE Healthcare, Waukesha, WI, USA) in the presence of 20 mM imidazole. The beads were washed three times with washing buffer (20 mM Tris–HCl pH 7.5, 500 mM NaCl) containing 40 mM imidazole. His-HN proteins were then eluted with washing buffer containing 8 M Urea, 500 mM imidazole. Amicon Ultra centrifugal filters (Millipore, Bedford, MA, USA) were used to concentrate the purified His-HN proteins by approximately 10- to 20-fold. The protein concentration was determined using the Bradford method with bovine serum albumin (BSA) as a protein standard.

### IMMUNIZATIONS AND GENERATION OF HYBRIDOMAS

Monoclonal antibodies specific for HPIV3-HN were generated using the previously described hybridoma technology (Kimura et al., 1994). In brief, 300 μg of N-terminal, His-tagged full-length HPIV3-HN protein was injected into the footpad of Balb/c mice using keyhole limpet hemocyanin as an adjuvant. Four weeks later, spleen cells were isolated and fused to the myeloma cell line SP2/O using polyethylene glycol 1500 (PEG 1500) as previously described (Kimura et al., 1996). Isotype determination was performed with the mouse MAb isotyping test kit (Bio-Rad, Hercules, CA, USA) according to the manufacturer’s protocol.

### ELISA

Microtiter plates coated with HPIV3-HN were incubated with threefold serial dilutions of each antibody (starting from 1:300 dilution of a hybridoma culture supernatant). After incubation with a peroxidase-conjugated secondary antibody and washing with PBS, the colorimetric signal of tetramethylbenzidine was detected by measuring the absorbance at 405 nm (Abs) using a plate reader.

### IMMUNOBLOTTING

Recombinant HPIV3-HN proteins (equivalent to ~100 ng) or HPIV3-infected HeLa cell lysates were separated by 10% SDS-Gel and transferred onto a PVDF membrane (Millipore). The membrane was then soaked in Tris-buffered saline (TBS) containing 5% (w/v) skim milk for 1 h and incubated with a MAb (hybridoma supernatant, 1:10 dilution) in TBS containing 0.1% (v/v) Tween-20 (TBST) overnight at 4°C. After washing three times with TBST, the membrane was incubated for 1 h in TBST containing goat-anti mouse IgG-HRP antibody (1:10000; GE Healthcare, Buckinghamshire, UK). After washing three times in TBST, the blot was detected with ImmobilonWestern Chemiluminescent HRP Substrate (Millipore) using FluorChem FC2 (Alpha Innotech, Santa Clara, CA, USA) in accordance to the manufacturer’s protocol.

### IMMUNOPRECIPITATION

HeLa cells were infected with HPIV3 (Strain C243) at multiplicity of infection (MOI) of 100 for 48 h. The cells were lysed with immunoprecipitation buffer (50 mM Tris-HCl pH 7.5, 100 mM NaCl, 0.5% NP-40, 200 μM PMSF, 50 μM VO_4_, 2 μg/ml Aprotinin, 5 μg/ml Leupeptin, 1 μg/ml Pepstatin A). For immunoprecipitation assay, cell lysate or wheat germ cell extract generating full-length HPIV3-HN was incubated with the individual MAb (hybridoma supernatant), preclearing overnight using proteinA/G sepharose beads, for 2 h at 4°C. After washing three times with immunoprecipitation buffer, immunocomplexes were eluted from the beads with 2x SDS sample buffer. Then, the bound protein was analyzed by immnoblotting.

### IMMUNOFLUORESCENCE

HeLa cells were grown on coverslips for 24 h and the cells were infected with HPIV3 (MOI = 100); mock-infected cells served as the control. At 48 h post-infection, the cells were washed with PBS before fixation with 3% formalin in PBS at room temperature for 15 min. Cells were washed twice with PBS for 5 min followed by 100% methanol for 10 min at -20°C. Cells were permeabilized with PBS containing 0.05% Triton X-100 for 10 min at room temperature. The cells were incubated with individual primary antibodies for 1 h at room temperature. After being washed twice with PBS, the cells were incubated with secondary antibodies for 1 h at room temperature. The nucleus was counterstained with 4′,6-diamidino-2-phenylindole (DAPI). Microscopic imaging was performed with an FV1000-D confocal laser scanning microscope (Olympus, Tokyo, Japan) equipped with a 60x oil-immersion objective.

### FLOW CYTOMETRY

HeLa cells were seeded in 6-well plates at a concentration of 2 × 10^5^ per well 24 h before infection, and the cells were washed with medium containing 0.0001% trypsin and infected with HPIV3 (MOI = 100); mock-infected cells were used as a control. At 4 days post-infection, cells were harvested in PBS containing 5 mM EDTA and washed twice with PBS. The cells were then incubated with either MAbs (hybridoma supernatant; 5X dilution) or non-immunized hybridoma supernatant in PBS containing 2% Blocking One (NACALAI TESQUE, INC., Kyoto, Japan) for 1 h at room temperature. After being washed twice with PBS, the cells were incubated with phycoerythrin (PE)-conjugated anti-mouse IgG antibodies (Beckman Coulter, Fullerton, CA, USA) for 1 h at room temperature. Flow cytometric analysis of the cells (10,000 cells per sample) was performed on a FACScanto II instrument (BD Biosciences, San Jose, CA, USA).

### EPITOPE MAPPING AND SPECIFICITY OF MAbs USING ALPHASCREEN ASSAY

The AlphaScreen assay was performed using 384-well ProxiPlates (PerkinElmer, Boston, MA, USA). Biotinylated virus proteins or GST (negative control) were incubated with a 40-fold dilution of MAbs (hybridoma supernatant) in 15 μl of binding mixture containing reaction buffer (100 mM Tris-HCl, pH 7.5, 1 mg/ml BSA, 0.01% Tween-20) at 26°C for 30 min. Then, combine 10 μl of the detection mixture containing 0.1 μl protein G-conjugated acceptor beads and 0.1 μl streptavidin-coated donor beads (AlphaScreen IgG detection kit, PerkinElmer) in reaction buffer were incubated at 26°C for 1 h. Antigen-antibody interactions were analyzed using an Envision microplate reader (PerkinElmer).

### PROTEOMIC ANALYSIS

Cell lysate from HPIV3-infected or mock-infected HeLa cells were immunoprecipitated with #21 MAb. The binding proteins were separated by SDS-PAGE and transferred to PVDF membranes. For LC-MS/MS analysis, the membranes digested with trypsin. LC-MS/MS analysis was performed using a TripleTOF MS (TripleTOF 5600 system, AB SCIEX, Foster City, CA, USA) and the Analyst version 1.6 TF (AB SCIEX) coupled to an DiNa-AP (KYA Technologies, Tokyo, Japan). Prior to injection into the mass spectrometer, the tryptic digests were filtered through a Ultrafree-MC, GV 0.22 μm filter (Millipore), then loaded onto a reverse phase pre-column (HiQ sil C18W-3, 500 μm id × 1 mm, KYA Technologies) and resolved on a nanoscale HiQ sil C18W-3 (100 μm id × 10 cm; KYA Technologies) at a flow rate of 200 nL/min with a gradient of acetonitrile/0.1% (v/v) formic acid. Peptides were separated using a 30 min gradient from 5 to 100% solvent B [0.1% (v/v) formic acid/80% (v/v) acetonitrile]. Solvent A was 0.1 % (v/v) formic acid/2% (v/v) acetonitrile. The obtained MS and tandem-MS data were searched against the human protein sequences in the Swiss-Prot database (version Jan 2013, 20233sequences) using the Protein Pilot software 4.5 (AB SCIEX).

## RESULTS

### PRODUCTION OF MAbs

**FIGURE 1:**
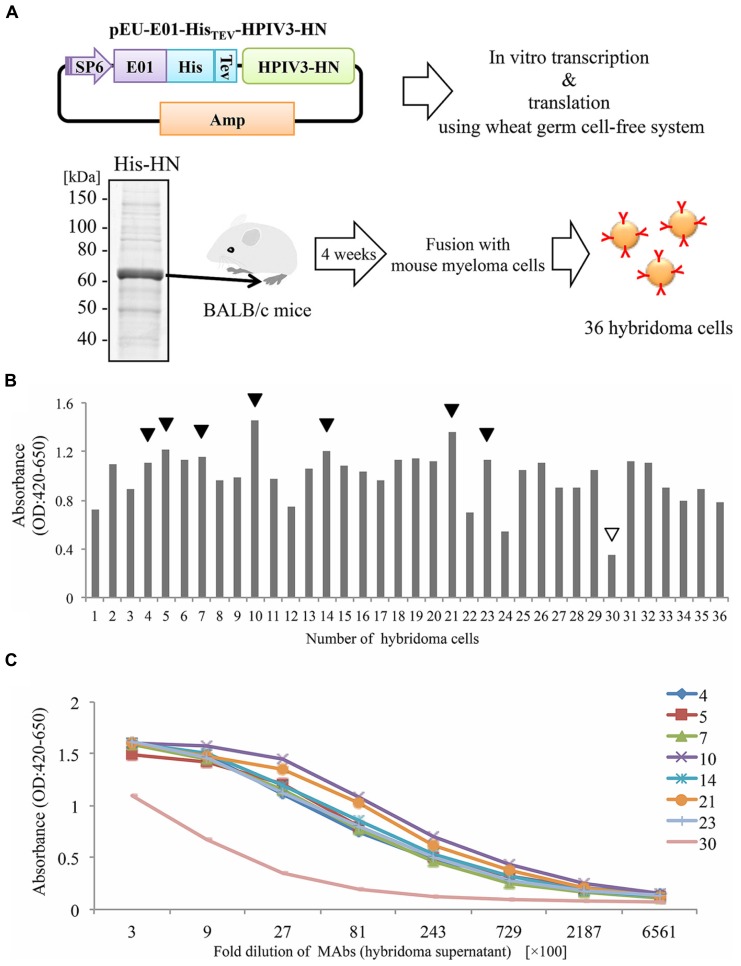
**Production of hybridoma cells generating anti-HPIV3-HN antibodies. (A)** Schematic diagram of hybridoma cells production generating anti-HPIV3-HN monoclonal antibody (MAb). The recombinant Histidine-tagged recombinant HPIV3-HN (His-HN) protein was produced by wheat germ cell-free system and then purified by nickel-chelated sepharose beads in the presence of 8 M urea. The purified protein was separated by SDS-PAGE and visualized by CBB-staining. Purified His-HN protein was injected into the footpad of Balb/c mice. After 4 weeks, immunized mouse splenocytes were fused with myeloma cells and then 36 hybridoma cells were established. SP6, SP6 promoter sequence; E01, translation enhancer sequence; His, Histidine-tagged sequence; TEV; TEV protease recognized sequence. **(B,C)** The specificity of MAbs (hybridoma supernatant) evaluated by ELISA. The specificity of 36 MAbs in 2700-fold dilution was determined **(B)**. The black arrows indicate the selected MAbs while the white arrow depicts a selected clone as a negative control (clone no. #30). The selected eight MAbs were diluted at serial points and analyzed by ELISA **(C)**.

In our current study, we attempted to produce the full-length HPIV3-HN by the wheat cell-free production system (**Figure 1A**). Complementary DNA encoding HPIV3-HN open-reading frame was sub-cloned into pEU-His, the expression vector designed specifically for the wheat germ cell-free system for expressing His-tagged protein. Consequently, His-tagged HPIV3-HN protein was synthesized by this procedure in a large scale. Since HPIV3-HN exhibited high insolubility in regular buffer, the protein was suspended in the buffer including 8 M urea. This suspended His-tagged HPIV3-HN was further purified using Ni-sepharose followed by the elusion with imidazole. Balb/c mice were then immunized with purified full-length HPIV3-HN protein. After 4 weeks, splenocytes were isolated and hybridomas were created (**Figure 1A**). Finally, 36 stable hybridomas were obtained and designated #1 to #36. The resulting hybridomas were screened by ELISA with plates coated with HPIV3-HN protein conjugated with BSA. Of the 36 hybridoma clones established, seven clones (#4, #5, #7, #10, #14, #21, #23) exhibited relatively high absorbances (**Figure 1B**). Titration analyses with diluted hybridoma supernatants or antigenic HN protein revealed that these seven antibodies had higher specificities and intensities than control hybridoma supernatants (**Figure 1C**). These seven hybridomas were processed for further characterization.

### IMMUNOBLOTTING ANALYSES OF MAbs

**FIGURE 2:**
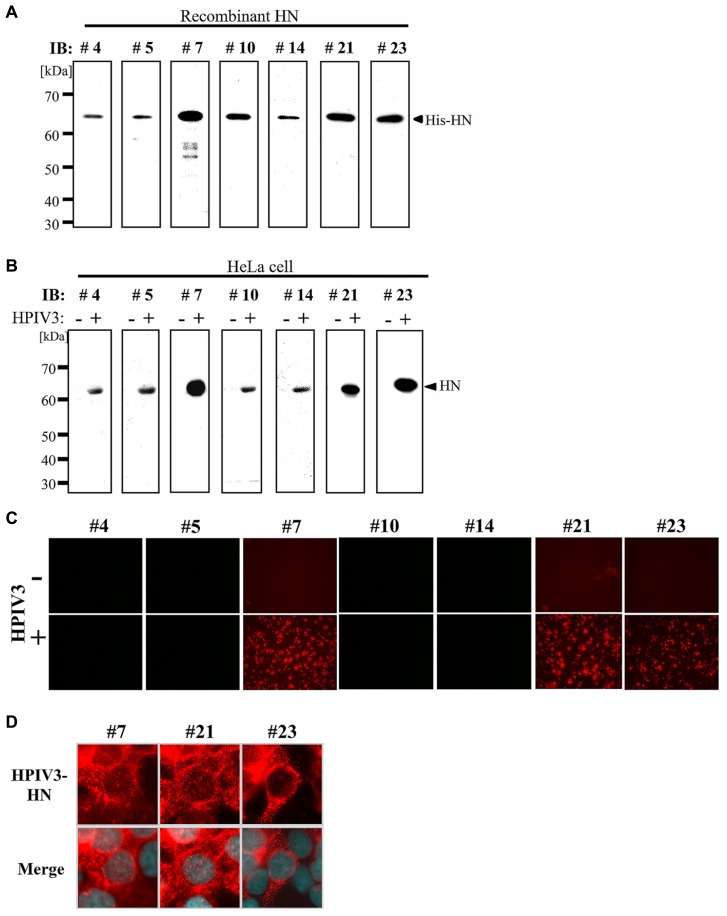
**Immunoblotting and immunofluorescent analysis. (A,B)** Detection sensitivity of the MAbs for recombinant His-HN **(A)** or HPIV3-infected cell lysate **(B)**. Recombinant HPIV3-HN (100 ng) was separated using 12.5% SDS-gel and transferred to a PVDF membrane, followed by incubation with MAbs (hybridoma supernatants) at a 1:10 dilution **(A)**. HeLa cells were infected or mock-infected with HPIV3. After 48 h, cells were lysed with SDS-PAGE loading buffer. The total protein was separated in 12.5% SDS-gel and immunobloted with indicated MAbs **(B)**. **(C,D)** Immunofluoresent analysis of HN (red) in HPIV3-infected HeLa cells. HeLa cells were infected or mock-infected with HPIV3. After 48 h, cells were fixed, and then stained with MAbs (hybridoma supernatant; red) and DAPI (blue). Confocal microscopic analysis was performed at 40× **(C)** and at 600× magnifications **(D)**.

We next tested the MAbs in immunoblotting analysis. First, recombinant HPIV3-HN protein was separated by SDS-PAGE followed by immunoblotting with MAbs isolated from the seven hybridomas. As shown in **Figure 2A**, all seven MAbs recognized a single band that corresponded to the recombinant protein. Next, immunoblotting analysis was performed with cell lysates from HeLa cells either infected- or mock-infected with HPIV3. The all seven MAbs detected a 63 kDa protein band that was consistent with the molecular mass of the HN protein (**Figure 2B**). No other bands were detected by the MAbs indicating that they specifically recognized HN.

### IMMUNOFLUORESCENT ANALYSIS

We next performed an immunofluorescent (IF) analysis of HPIV3-infected HeLa cells with seven MAbs (**Figure 2C**). Three of the MAbs (#7, #21, and #23) exhibited prominent IF staining of the infected cells, while the other four MAbs had no detectable IF staining. The IF staining with the antibodies revealed that HN protein exhibited granular staining pattern throughout cytoplasm and plasma membrane in HPIV3-infected cells (**Figure 2D**), which was consistent with previous studies (Ali and Nayak, 2000; Stone and Takimoto, 2013). The control mock-infected cells did not show any signals when stained with the antibodies (**Figure 2C**). We thus selected the three MAbs (#7, #21, and #23) for further characterization.

### IMMUNOPRECIPITATION

**FIGURE 3:**
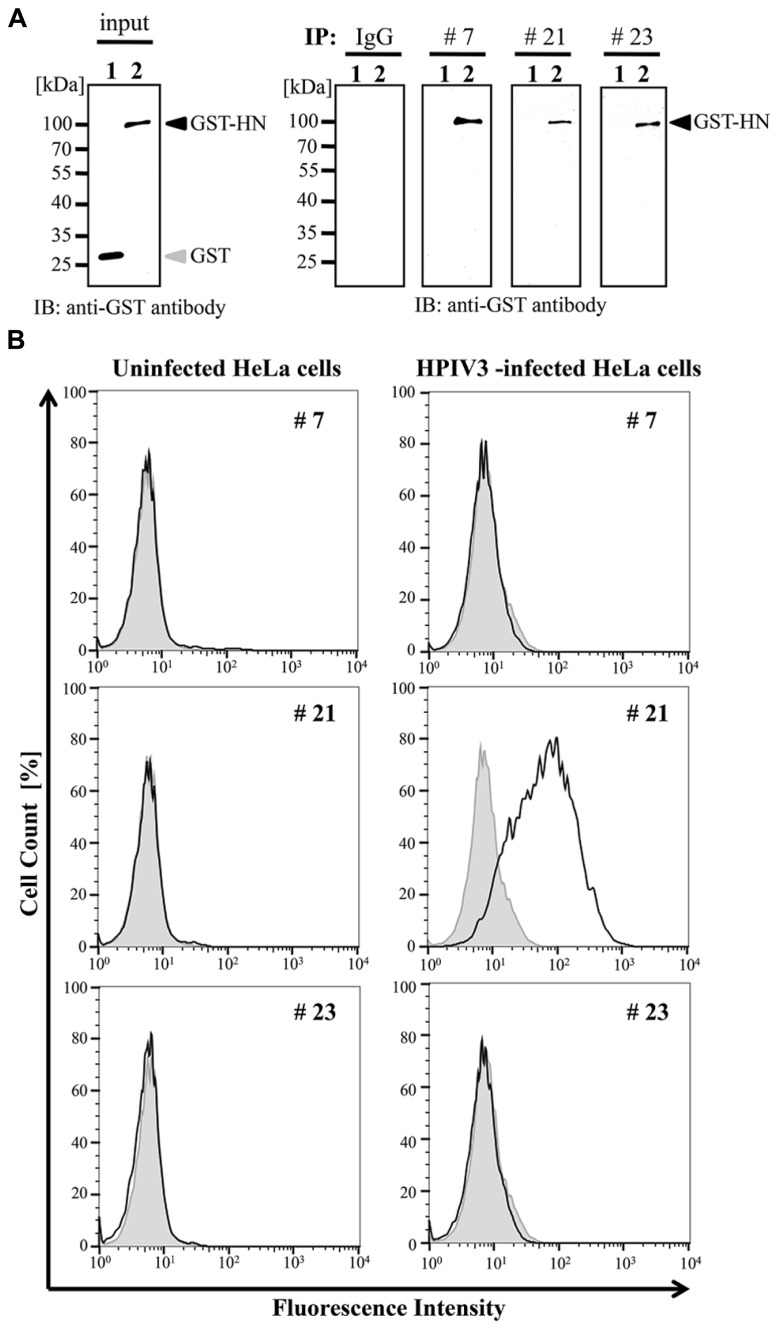
**Immunoprecipitation and flow cytometry assay. (A)** Recombinant GST-HPIV3-HN or GST protein was immnoprecipitated with either #7, #21, #23 MAbs, or IgG (negative control), respectively. Then bound proteins were analyzed with immunoblotting using anti-GST antibody. **(B)** HPIV3-infected or uninfected HeLa cells were harvested at 4 days post-infection, followed by incubation with indicated MAbs. The cells were then fixed and stained with anti-mouse secondary antibody. The population of stained cells was calculated by flow cytometry. The shaded histogram shows negative hybridoma supernatant and the bold line shows specific MAbs.

We next examined whether these selected antibodies were useful in immunoprecipitation analysis. The wheat germ extract containing full-length HPIV3-HN with glutathione-*S*-transferase (GST) tag was incubated with protein A/G-coated sepharose beads (GE Helthcare) together with the three selected antibodies or non-immunized mouse IgG antibody. The precipitated samples were subjected to immunoblotting analysis with anti-GST antibody. The GST tagged HN protein was precipitated by all selected antibody (#7, #21, and #23) but not non-immunized IgG (**Figure 3A**).

### FLOW CYTOMETRY ANALYSIS

We next addressed the usability of the antibodies #7, #21, and #23 in flow cytometry analysis. HeLa cells infected with HPIV3 were stained with the antibodies and then subjected to FACS analysis. Our results demonstrated that only #21 MAbs could specifically detect virus-infected cell populations, and distinguished between viral-infected and uninfected cell population with flow cytometory (**Figure 3B**).

### EPITOPE MAPPING OF MAbs

**FIGURE 4:**
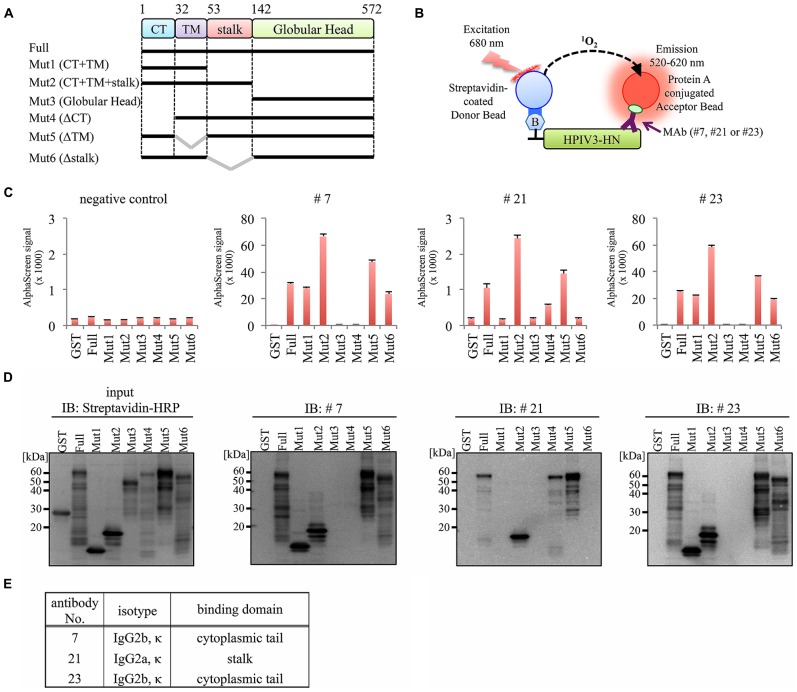
**Epitope mapping for generated antibodies. (A)** Schematic diagram of seven deletion mutants of HPIV3-HN for epitope mapping. These proteins were produced as N-terminal biotinylated protein by wheat germ cell-free system. **(B)** Schematic diagram of the AlphaScreen assay used to detect the binding of MAb to full-length or deletion mutants of HPIV3-HN. The interaction between antibodies and recombinant proteins was monitored by AlphaScreen with protein A-conjugated acceptor beads and streptavidin-coated donor. Upon excitation at 680 nm, singlet oxygen molecules were produced from the donor beads, which reacted with the acceptor beads, resulting in light emission that was measured between 520 and 620 nm. **(C)** In AlphaScreen assay, the binding activity was measured as the level of the AlphaScreen luminescence signal. Error bars represent standard deviations from three independent experiments. **(D)** The biotinylated-full-length HN, its deletion mutants and GST proteins were separated by SDS-PAGE and transferred to PVDF membrane, followed by immunoblotting with Strepavidin-HRP andibody (left panel) and indicated MAb (right panel). **(E)** Summary of properties of selected MAbs. Immunoglobulin isotyping was carried out with mouse monoclonal antibody isotyping test kit.

To determine the binding domain of the MAbs within the HPIV3-HN protein, we synthesized six different deletion mutants of HPIV3-HN as depicted in **Figure 4A**. All of the deletion mutants contained N-terminal biotin tag were incubated with the antibodies (#7, #21, or #23), followed by the addition of AlphaScreen streptavidin donor and protein A acceptor beads, as depicted in **Figure 4B**. The reactivity was measured and calculated by the level of the AlphaScreen luminescent signal. The results showed that two MAbs (#7 and #23) reacted against the cytoplasmic tail (CT) of HPIV3-HN whereas #21 MAb detected extracellular stalk region of the protein (**Figures 4C–E**). This result is fully consistent with the result of flow cytometry analysis (**Figure 3B**). 

### SPECIFICITY OF MAb

**FIGURE 5:**
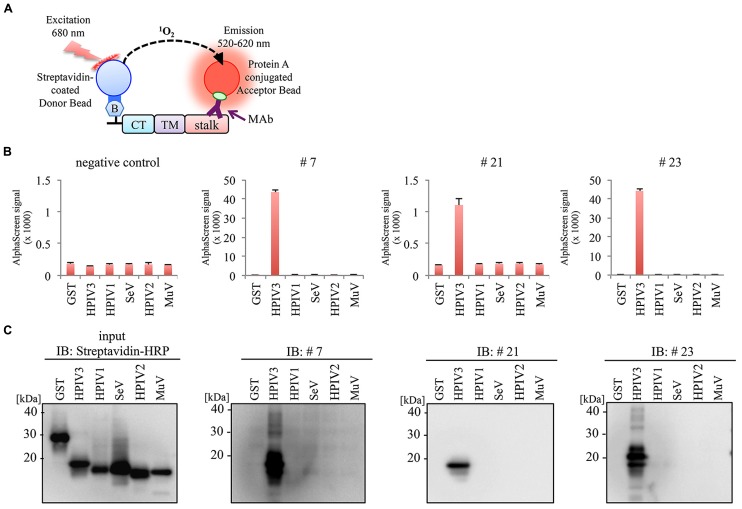
**No evident cross-reactivity of selected MAb to other paramyxoviruses. (A)** Schematic diagram of AlphaScreen assay. The biotinylated partial HN proteins derived from several paramyxoviruses containing CT, TM and stalk region were produced by wheat germ cell-free system. **(B,C)** Specificity of MAbs was varidated by AlphaScreen assay **(B)** and immunoblotting **(C)**. In AlphaScreen assay, the binding activity was measured as the level of the AlphaScreen luminescence signal **(B)**. Error bars represent standard deviations from three independent experiments. The biotinylated partial HN proteins were separated by SDS-PAGE and transferred to PVDF membrane, followed by immunoblotting with either Strepavidin-HRP andibody (left panel) or anti-HN MAbs (right panel; **C**).

We next investigate the specificity of MAbs using HN protein frangments derived from HPIV1, Sendai virus (SeV), HPIV2, and Mumpus virus (MuV). Partial HN protein fragments containing cytoplasmic tail (CT), transmembrane domain (TM), and stalk region were produced with a N-terminal biotin tag by wheat cell-free system, and then incubated with the antibodies (#7, #21, or #23) followed by the AlphaScreen (**Figure 5A**). The antigen reactivity was measured based on the level of the AlphaScreen luminescent signal (**Figure 5B**). Notably, there was no cross-reactivity to HN proteins derived from other Paramyxoviruses except for HPIV3. This was also confirmed by immunoblotting analysis (**Figure 5C**). These results indicate the specificity of the antibodies for HPIV3-HN.

### PROTEOMIC ANALYSIS

**FIGURE 6:**
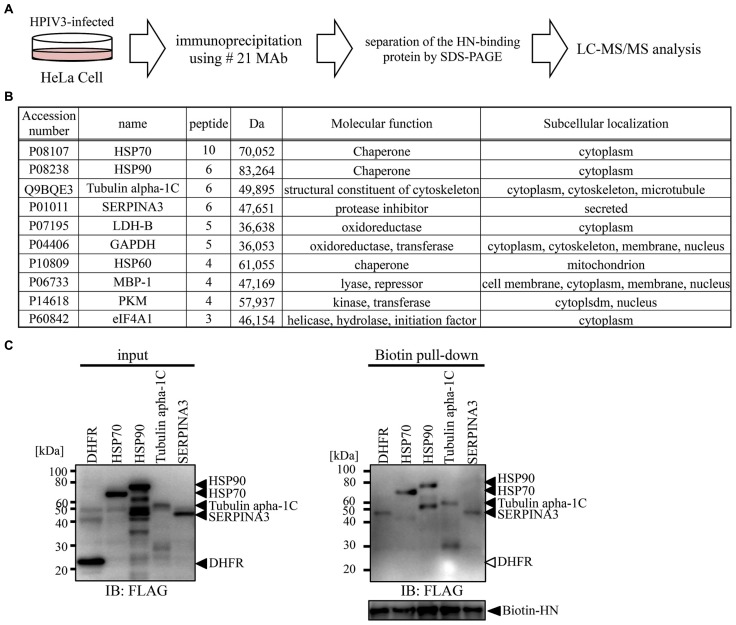
**Proteomics analysis using selected MAb. (A)** Schematic diagram of proteomic analysis for the identification of PIV3-HN-binding protein. HPIV3-infected HeLa cell lysate was immunoprecipitated with #21 MAb. The bound proteins were separated by SDS-PAGE and analyzed by LC-Ms/Ms. **(B)** The panel shows the list of putative HN-binding proteins identified by mass spectrometry analysis. **(C)** FLAG-tagged HSP70, HSP90, tubulin alpha 1C, or SERPINA3 proteins were mixed with HPIV3-HN. Samples were pull-down with the streptavidin magnetic beads and the collected proteins were separated by SDS-PAGE. The bound protein detected by immunoblotting analysis with anti-FLAG antibody. The right arrows indicated the position of each protein.

We next utilized our newly developed #21 MAb for the identification of host proteins that bind to HPIV3-HN during HPIV3 infection. Cell lysate from HPIV3-infected or mock-infected cells were immunoprecipitated with #21 MAb. Precipitated samples were collected and then digested with trypsin followed by LC-MS/MS analysis (**Figure 6A**). Annotation analysis using the Swiss-Prot database revealed that 10 proteins were putative HN binding proteins (**Figure 6B**). Based on the number of corrected peptides, top four proteins (HSP70, HSP90, tubulin, alpha 1c, and SERPINA3) were selected as the most likely candidates for association with HN and subjected to further binding analysis. The pull-down analysis of the host proteins with recombinant HPIV3-HN was performed. The subsequent immunoblotting analysis demonstrated that HPIV3-HN could indeed interact with these four proteins (**Figure 6C**). These results demonstrate the availability of our newly-developed antibody in comprehensive proteomic analysis.

## DISCUSSION

Herein we produced HPIV3-HN proteins by the wheat cell-free system, and created MAbs that selectively target the HPIV3-HN protein. Characterization of the most potent MAb confirmed the antigen-specificity and usability in various applications including immunoblotting, immnofluorescent, flow cytometry, and immunoprecipitation analyses. Furthermore, the MAb could capture the endogenous HN protein from HPIV3-infected cells to identify HN-binding host proteins via mass spectrometry-based proteomic analysis. Our current results demonstrated the generation of useful antibody against HPIV3-HN and also shed new light on the unexplored molecular link between the PIV3-HN and host proteins.

Currently, cell-based production (e.g., *E. coli* system or baculovirus-insect cell system) of recombinant virus proteins has been widely used. However, it is often difficult to produce sufficient quantities of viral antigens in the conventional cell-based system because many viral antigens are usually insoluble, cytotoxic, and are expressed in the inclusion body fraction. In contrast, the cell-fee protein production system enables the synthesis of toxic proteins that are otherwise excluded from production in live cells. Among the cell-free approaches, the wheat germ cell-free system employs a eukaryotic translation system that warrants the synthesis of properly folded and biologically active proteins similar to proteins that are expressed in living mammalian cells (Endo and Sawasaki, 2005, 2006). These advantages underscore the suitability and availability of the wheat germ cell-free system for the generation of antigenic proteins that can be used for animal immunizations to generate MAbs. Our current study clearly demonstrated the benefit of using viral proteins synthesized by the wheat germ cell-free system to efficiently produce the MAbs against the viral antigen. Using this approach, we have created MAbs against HPIV3-HN that detected both denatured and native forms of the antigen. These MAbs were useful in various immunological assays including ELISA, IF, immunoblotting, and immunoprecipitation. Further careful studies of structural aspects are needed to determine whether the MAb can affect the virus infectivity.

We identified Hsp70 as a putative HN binding protein. Several previous studies demonstrated that Hsp70 was involved in the regulation of other RNA viruses. Hsp70 is known to associate with viral PB1 and PB2 subunits of influenza A virus, and it negatively regulated the expression of viral proteins in infected cells (Li et al., 2011). In another study, Hantavirus infection induced the expression of HSP70 that interacted with nucleocapsid protein and its overexpression suppressed viral infection in Vero E6 cells (Yu et al., 2009). In contrast, Hsp70 was found to positively regulate rabies viral infection. Indeed, rabies infection induced the cellular expression of Hsp70 and accumulation in Negri body-like structures, which are the site of viral transcription and replication. Inhibition of Hsp70 resulted in a significant decrease of viral mRNAs, viral proteins, and virus particles (Lahaye et al., 2009, 2012). Taken together these results indicated a pivotal role of Hsp70 in viral replication and the pathogenicity of viral infection. Hsp70 binds and regulates many cellular proteins, as well as viral proteins (Pratt and Toft, 2003; Mayer, 2005; Silver and Noble, 2012), and the effects of Hsp70 on viral infection are diverse and unique between different viral species or cell systems. Further studies are required to investigate the precise molecular mechanism by which the association of Hsp70 and HN proteins mediate HPIV3 replication.

We also found that PIV3-HN can interact with Serpin3a. Serpin3a, as also known as alpha-1-antichymotrypsin, is a member of serpin proteins involved in the inhibition of serine and other types of proteases (Bauman et al., 2002). In humans, the majority of serpins regulates the functions of proteases involved in the response against body’s injuries such as coagulation, fibrinolysis, inflammation, wound healing and tissue repair. Serpins have been also implicated in various pathologies in respiratory system such as airway hyperresponsiveness (AHR) and asthma (Sivaprasad et al., 2011). The physical interaction of HPIV3-HN with serpin3a may be involved in hindering the function of Serpin3a toward respiratory disorders. Further analysis may shed new light on understanding the etiology of HPIV3-induced asthma.

In summary, we utilized the wheat cell-free production system to create and characterize MAbs that may be useful in various immunological applications. Our newly-developed MAbs could thus provide a valuable means to explore HPIV3 infection in human cells.

## Conflict of Interest Statement

The authors declare that the research was conducted in the absence of any commercial or financial relationships that could be construed as a potential conflict of interest.
